# Crystal structure and Hirshfeld surface analysis of (2^2^ *RS*,2^3^ *SR*,2^5^ *RS*,2^6^ *SR*)-2^3^,2^5^,5-trimethyl-2^1^-(2,2,2-tri­fluoro­acet­yl)-5-aza-2(2,6)-piperidina-1,3(2,5)-di­furana­cyclo­hexa­phan-2^4^-one

**DOI:** 10.1107/S2056989023001986

**Published:** 2023-03-10

**Authors:** Sema Öztürk Yıldırım, Mehmet Akkurt, Anastasia A. Ershova, Mikhail S. Grigoriev, Bruno G.M. Rocha, Ajaya Bhattarai

**Affiliations:** a Department of Physics, Faculty of Science, Eskisehir Technical University, Yunus Emre Campus 26470 Eskisehir, Türkiye; b Department of Physics, Faculty of Sciences, Erciyes University, 38039 Kayseri, Türkiye; c Peoples’ Friendship University of Russia (RUDN University), 6 Miklukho-Maklaya St., Moscow, 117198, Russian Federation; d Frumkin Institute of Physical Chemistry and Electrochemistry, Russian Academy of Sciences (IPCE RAS), 31 Bldg 4, Leninsky prosp., Moscow, 119071, Russian Federation; e Centro de Química Estrutural, Institute of Molecular Sciences, Instituto Superior Técnico, Universidade de Lisboa, Av. Rovisco Pais, 1049-001 Lisboa, Portugal; f Department of Chemistry, M.M.A.M.C (Tribhuvan University), Biratnagar, Nepal; University of Neuchâtel, Switzerland

**Keywords:** crystal structure, twelve-membered heterocycles, furan, alkyl­ation, piperidon, Hirshfeld surface analysis, Mannich reaction

## Abstract

The title compound, C_20_H_21_F_3_N_2_O_4_, features a main twelve-membered difuryl ring with which the furan rings make dihedral angles of 76.14 (5) and 33.81 (5)°. The dihedral angle between the furan rings is 42.55 (7)°. The six-membered nitro­gen heterocycle has a twist-boat conformation. In the crystal, pairs of mol­ecules are connected by inter­molecular C—H⋯O inter­actions, generating an *R* ^2^_2_(14) ring motif. These pairs of mol­ecules form *zigzag* chains along the *a*-axis direction by means of C—H⋯F inter­actions. Furthermore, C—H⋯π and C–F⋯π inter­actions link the mol­ecules into chains along the *b-*axis direction, forming sheets parallel to the (001) plane. These sheets are also connected by van der Waals inter­actions.

## Abstract 2

The title compound features a main twelve-membered difuryl ring with which the furan rings make dihedral angles of 76.14 (5) and 33.81 (5)°. In the crystal, C–H⋯O, C—H⋯F, C—H⋯π and C—F⋯π inter­actions link the mol­ecules into the chains along the *b-*axis direction, forming sheets parallel to the (001) plane. These sheets are also connected by van der Waals inter­action.

## 1. Chemical context

Twelve-membered aza- and oxa-macrocycles possess a wide range of useful biological activities and exhibit a tendency to bind metal cations with their macrocyclic cavities (Simonov *et al.*, 1993 ▸). For example, well-known naturally occurring macrocycles such as enniatins demonstrate a high cytotoxic activity (Levy *et al.*, 1995 ▸; Ivanova *et al.*, 2006 ▸) and aza­tri(tetra­)pyrrolic macrocycles can be used as ion-pair receptors (Yadigarov *et al.*, 2009 ▸). Chiral macrocycles with multiple non-covalent bonding sites show chiral recognition to different anions (Ema *et al.*, 2014 ▸; Khalilov *et al.*, 2021 ▸; Maharramov *et al.*, 2010 ▸). S,N-Containing macrobicyclic aza­cryptands (Khabibullina *et al.*, 2018 ▸; Naghiyev *et al.*, 2020 ▸; Safavora *et al.*, 2019 ▸) including dipyrrolyl­methane subunits in their structures exhibit a high affinity to anions, especially the fluoride ion (Guchhait, *et al.*, 2011 ▸; Shikhaliyev *et al.*, 2018 ▸, 2019 ▸) and can be used as chemical delivery systems.

**Figure 1:**
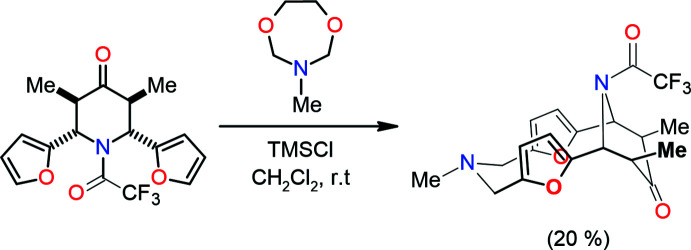
The synthetic route.

On the other hand, the Mannich reaction is an extensively used method for the construction of various types of polycyclic systems (Rivera *et al.*, 2015 ▸; Ma *et al.*, 2021 ▸; Mahmoudi *et al.*, 2016 ▸), including those containing pyrroles (Jana *et al.*, 2019). In order to create a short pathway to macrocycles possessing two different donating atoms in a twelve-membered ring, we used an acid-catalysed Mannich type reaction between 2,6-difuryl-substituted piperidone and *N*-substituted 1,5,3-diox­azepane (Fig. 1 ▸). The main goal of this study was to obtain the first representative of a twelve-membered difuryl containing rings and to establish its stereochemistry and non-covalent bond donor or acceptor ability (Gurbanov *et al.*, 2020*a* ▸,*b* ▸; Mahmudov *et al.*, 2021 ▸, 2022 ▸). The rings formed in this transformation can serve as precursors for studying the IMDAV (intra­molecular Diels–Alder reaction of vinyl­arenes; Krishna, *et al.*, 2022 ▸) and IMDAF (intra­molecular Diels–Alder reaction of furans; Kvyatkovskaya *et al.*, 2021*a* ▸,*b* ▸; Borisova, *et al.*, 2018 ▸) reactions. 
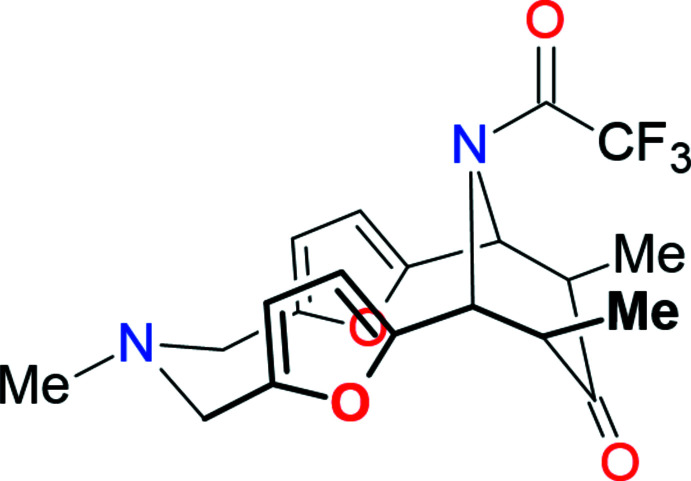


## 2. Structural commentary

**Figure 2:**
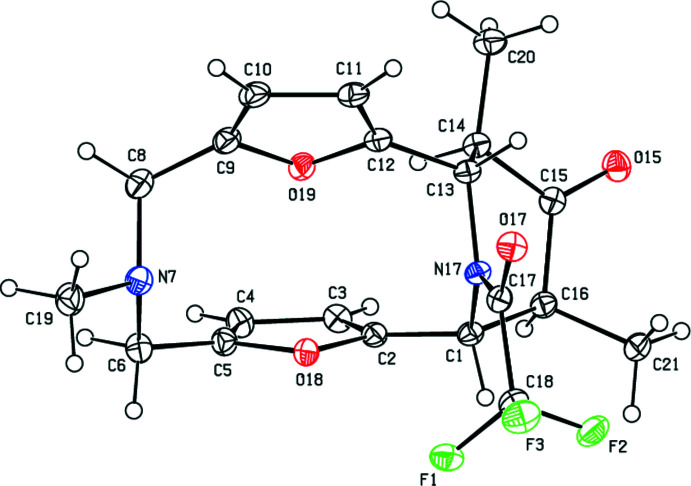
Mol­ecular structure of the title compound. Displacement ellipsoids are drawn at the 30% probability level.

As shown in Fig. 2 ▸, the title compound has a main twelve-membered difuryl-containing ring (O18 /C2/C1/N17/C13/C12/O19/C9/C8/N7/C6/C5) to which the furan rings (O18/C2–C5 and O19/C9–C12) subtend dihedral angles of 76.14 (5) and 33.81 (5)°, respectively. The dihedral angle subtended by the furan ring is 42.55 (7)°. The six-membered nitro­gen heterocycle (N17/C1/C13–C16) adopts a twist-boat conformation with puckering parameters (Cremer & Pople, 1975 ▸) *Q*_T_ = 0.6999 (12) Å, θ = 90.12 (10)° and φ = 228.08 (10)°.

## 3. Supra­molecular features and Hirshfeld surface analysis

**Figure 3:**
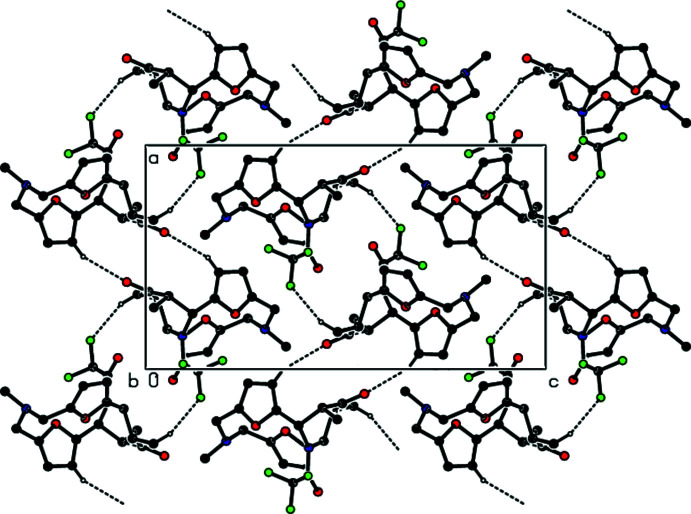
View down the *b*-axis showing the C—H⋯O and C—H⋯F hydrogen bonds (dashed lines).

**Figure 4:**
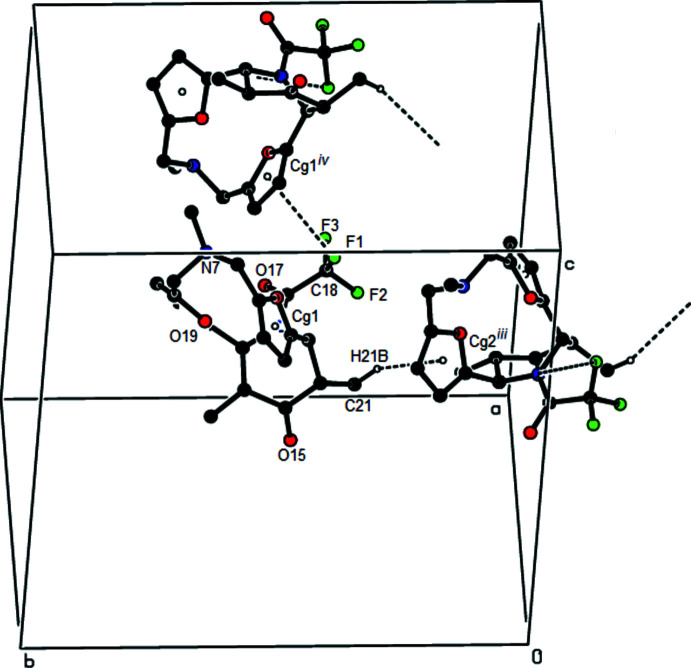
A general view in the unit cell of the C—H⋯π and C—F⋯π inter­actions (dashed lines). Symmetry codes: (iii) −*x* + 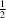 , *y* − 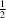 , *z*; (iv) 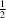 + *x*, *y*, 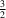 − *z*.

In the crystal, pairs of mol­ecules are connected by inter­molecular C—H⋯O inter­actions, forming an 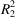 (14) ring motif (Bernstein *et al.*, 1995 ▸). These pairs of mol­ecules form zigzag chains along the *a*-axis direction by C—H⋯F inter­actions (Table 1 ▸, Fig. 3 ▸). Furthermore, C—H⋯π and C—F⋯π inter­actions [C18—F1⋯*Cg*1^i^, C18⋯*Cg*1^i^ = 3.9574 (14) Å, F1⋯*C*g1^i^ = 3.5265 (9) Å, C18—F1⋯*Cg*1^i^ = 98.83 (6)° and C18—F3⋯*Cg*1^i^, C18⋯*Cg*1^i^ = 3.9574 (14) Å, F1⋯*C*g1^i^ = 3.5496 (11) Å, C18—F1⋯*Cg*1^i^ = 97.90 (7)° where *Cg*1 is the centroid of the O18/C2–C5 ring; symmetry code: (i) 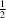 + *x*, *y*, 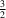 − *z*] link the mol­ecules into chains along the *b*-axis direction, forming sheets parallel to the (001) plane (Table 1 ▸, Fig. 4 ▸). These sheets are also connected by van der Waals inter­actions.

**Figure 5:**
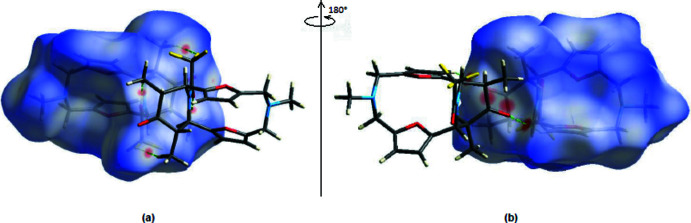
(*a*) Front and (*b*) back views of the three-dimensional Hirshfeld surfaces of the title mol­ecule.

*Crystal Explorer17.5* (Turner *et al.*, 2017 ▸) was used to perform a Hirshfeld surface analysis and to create the corres­ponding two-dimensional fingerprint plots, with the three-dimensional *d*_norm_ surfaces plotted at a standard resolution of −0.1525 (red) to 1.7277 (blue) a.u (Fig. 5 ▸). The bright-red patches near atoms O15 and H20*B* on the Hirshfeld surface represent weak C—H⋯O and C—H⋯F inter­actions (Tables 1 ▸ and 2 ▸).

**Figure 6:**
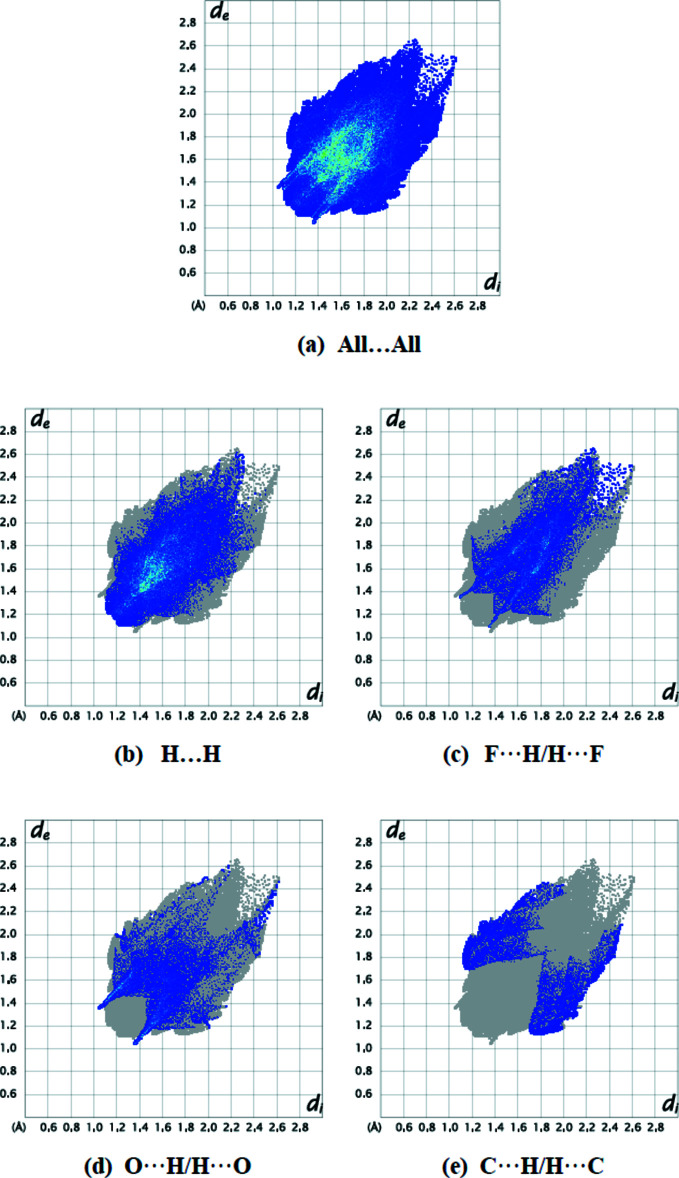
Two-dimensional fingerprint plots for title mol­ecules showing (*a*) all inter­actions, and delineated into (*b*) H⋯H, (*c*) F⋯H/H⋯F, (*d*) O⋯H/H⋯O and (*e*) C⋯H/H⋯C inter­actions. The *d*_i_ and *d*_e_ values are the closest inter­nal and external distances (in Å) from given points on the Hirshfeld surface.

The fingerprint plots (Fig. 6 ▸) show that H⋯H (44.9%), F⋯H/H⋯F (23.0%), O⋯H/H⋯O (16.7%) and C⋯H/H⋯C (8.5%) inter­actions contribute the most to surface contacts. The crystal packing is additionally influenced by F⋯C/C⋯F (3.0%), N⋯H/H⋯N (1.4%), F⋯O/O⋯F (0.9%), C⋯O/O⋯C (0.9%), O⋯O (0.5%) and C⋯C (0.1%) inter­actions. The Hirshfeld surface study confirms the significance of H-atom inter­actions in the packing formation. The large number of H⋯H, F⋯H/H⋯F, O⋯H/H⋯O and C⋯H/H⋯C inter­actions indicate that van der Waals inter­actions and hydrogen bonding are important in the crystal packing (Hathwar *et al.*, 2015 ▸).

## 4. Database survey

1,8,12,19,24,26-Hexaaza­penta­cyclo­[17.3.1.13,6.18,12.114,17]hexa­cosa-3,5,14,16-tetra­ene ethyl acetate solvate dihydrate (CSD refcode NOYCOW; Jana *et al.*, 2019 ▸) is the most similar compound to the title found in a search of the Cambridge Structural Database (CSD, Version 5.42, update of September 2021; Groom *et al.*, 2016 ▸). It crystallizes in the monoclinic space group *I*2/*a* (15) with *Z* = 8. The two pyrrolic NH atoms are oriented in the same direction. It exhibits a different conformation from the title compound: the furan rings in the title compound are almost normal to the mean plane of the main twelve-membered difuryl-containing ring and their oxygen atoms are oriented to the opposite sides whereas in NOYCOW, they are also almost normal, but are on the same side.

## 5. Synthesis and crystallization

A mixture of *N*-tri­fluoro-acyl­ated piperodone (2.6 mmol), 3-methyl-1,5,3-dioxazepane (2.7 mmol) and Me_3_SiCl (1.1 mL, 8.6 mmol) in dry di­chloro­methane (CH_2_Cl_2_) (5 mL) was left for 5 days under an argon atmosphere without stirring. The reaction mixture was then poured into water (30 mL) and basified with solid K_2_CO_3_ until the pH was 9–10. The organic products were extracted with CH_2_Cl_2_ (2 × 20 mL) and dried over anhydrous Na_2_SO_4_. After evaporation of the solvent, the crude residue was purified by column chromatography on silica gel (ethyl acetate/hexane, from 1:20 to 1:4) and then the resulting solid fractions were recrystallized from a chloro­form/hexane mixture to give the macrocycle as a white solid. Single crystals were obtained by slow crystallization from a hexa­ne/chloro­form mixture.

Yield 20% (0.21 g), m.p. 420–422 K. ^1^H NMR (700 MHz, CDCl_3_) δ (*J*, Hz): 6.24 (*br.s*, 2H), 6.19 (*br.s*, 1H), 6.02 (*d*, *J =* 2.9 Hz, 1H), 5.28 (*s*, 1H), 5.11 (*d*, *J =* 9.5 Hz, 1H), 3.86 (*d*, *J =* 15.5 Hz, 1H), 3.76 (*d*, *J =* 15.3 Hz, 1H), 3.71 (*s*, 2H), 3.55–3.49 (*m*, 1H), 3.20 (*q*, *J =* 7.2 Hz, 1H), 2.35 (*s*, 3H), 1.36 (*d*, *J =* 7.2 Hz, 3H), 1.08 (*d*, *J =* 6.4 Hz, 3H); ^13^C{^1^H} NMR (176 MHz, CDCl_3_) δ 208.8, 156.5 (*q*, *J* = 36.5 Hz), 154.7, 152.3, 149.9, 148.6, 116.3 (q, *J* = 289.0 Hz), 111.4, 109.9, 109.8, 109.6, 57.2, 56.9, 53.3, 49.6, 44.7, 42.8, 42.0, 15.7, 12.9; HRMS (ESI) *m*/*z*: [*M* + H]^+^ 411.; Analysis calculated for C_20_H_21_F_3_N_2_O_4_ %: C 58.53, H 5.16, N 6.83. Found: C 58.54, H 5.17, N 6.83.

## 6. Refinement

Crystal data, data collection and structure refinement details are summarized in Table 3 ▸. Carbon-bound H atoms were placed in calculated positions [C—H = 0.95–1.00 Å; *U*_iso_(H) = 1.2 or 1.5*U*_eq_(C)] and were included in the refinement in the riding-model approximation. Owing to poor agreement between observed and calculated intensities, twenty three outliers (0 0 6, 4 0 12, 5 1 6, 3 6 3, 4 8 5, 4 5 5, 12 11 0, 0 6 3, 4 7 5, 1 0 8, 1 1 2, 0 4 9, 6 5 2, 4 8 0, 3 6 7, 7 1 1, 4 1 9, 5 0 6, 0 0 2, 2 1 7, 4 2 8, 4 4 5, 2 5 5) were omitted during the final refinement cycle.

## Supplementary Material

Crystal structure: contains datablock(s) I. DOI: 10.1107/S2056989023001986/tx2063sup1.cif

Structure factors: contains datablock(s) I. DOI: 10.1107/S2056989023001986/tx2063Isup2.hkl

CCDC reference: 2245808

Additional supporting information:  crystallographic information; 3D view; checkCIF report

## Figures and Tables

**Table 1:** Hydrogen-bond geometry (Å, °) *Cg*2 is the centroid of the O19/C9–C12 ring.

*D*—H⋯*A*	*D*—H	H⋯*A*	*D*⋯*A*	*D*—H⋯*A*
C3—H3⋯O15^i^	0.95	2.51	3.3809 (15)	152
C20—H20*B*⋯F3^ii^	0.98	2.54	3.4700 (16)	160
C21—H21*B*⋯*Cg*2^iii^	0.98	2.88	3.7561 (13)	150

Symmetry codes: (i) 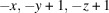 ; (ii) 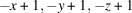 ; (iii) 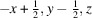 .

**Table 2:** Summary of short inter­atomic contacts (Å) in the title compound

N7⋯H4	2.71	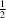 + *x*, *y*, 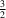 − *z*
F2⋯H19*C*	2.65	1 − *x*, − 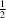 + *y*, 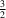 − *z*
H20*B*⋯F3	2.54	1 − *x*, 1 − *y*, 1 − *z*
H21*A*⋯H19*A*	2.46	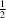 − *x*, 1 − *y*, − 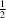 + *z*
H20*A*⋯H16	2.48	-*x*, 1 − *y*, 1 − *z*
H20*C*⋯H21*B*	2.50	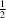 − *x*, 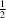 + *y*, *z*
H20*A*⋯H11	2.53	− 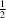 + *x*, 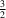 − *y*, 1 − *z*

**Table 3:** Experimental details

Crystal data	
Chemical formula	C_20_H_21_F_3_N_2_O_4_
*M*_r_	410.39
Crystal system, space group	Orthorhombic, *P* *b* *c* *a*
Temperature (K)	100
*a*, *b*, *c* (Å)	11.1351 (1), 17.0545 (2), 19.9131 (3)
*V* (Å^3^)	3781.57 (8)
*Z*	8
Radiation type	Cu *K*α
μ (mm^−1^)	1.03
Crystal size (mm)	0.25 × 0.20 × 0.20

Data collection	
Diffractometer	XtaLAB Synergy, Dualflex, HyPix
Absorption correction	Multi-scan (*CrysAlis PRO*; Rigaku OD, 2021 ▸)
*T*_min_, *T*_max_	0.761, 0.801
No. of measured, independent and observed [*I* > 2σ(*I*)] reflections	24690, 4040, 3752
*R*_int_	0.031
(sin θ/λ)_max_ (Å^−1^)	0.637

Refinement	
*R*[*F* ^2^ > 2σ(*F* ^2^)], *wR*(*F* ^2^), *S*	0.037, 0.102, 1.03
No. of reflections	4040
No. of parameters	265
H-atom treatment	H-atom parameters constrained
Δρ_max_, Δρ_min_ (e Å^−3^)	0.32, −0.21

Computer programs: *CrysAlis PRO* (Rigaku OD, 2021 ▸), *SHELXT* (Sheldrick, 2015*a* ▸), *SHELXL2018* (Sheldrick, 2015*b* ▸), *ORTEP-3 for Windows* (Farrugia, 2012 ▸) and *PLATON* (Spek, 2020 ▸).

## supplementary crystallographic information

### Crystal data

**Table:**

C_20_H_21_F_3_N_2_O_4_	*D*_x_ = 1.442 Mg m^−^^3^
*M**_r_* = 410.39	Cu *K*α radiation, λ = 1.54184 Å
Orthorhombic, *P**b**c**a*	Cell parameters from 15774 reflections
*a* = 11.1351 (1) Å	θ = 2.2–78.7°
*b* = 17.0545 (2) Å	µ = 1.03 mm^−^^1^
*c* = 19.9131 (3) Å	*T* = 100 K
*V* = 3781.57 (8) Å^3^	Prism, colourless
*Z* = 8	0.25 × 0.20 × 0.20 mm
*F*(000) = 1712	

### Data collection

**Table:**

XtaLAB Synergy, Dualflex, HyPix diffractometer	3752 reflections with *I* > 2σ(*I*)
Radiation source: micro-focus sealed X-ray tube	*R*_int_ = 0.031
φ and ω scans	θ_max_ = 79.3°, θ_min_ = 5.2°
Absorption correction: multi-scan (CrysAlisPro; Rigaku OD, 2021)	*h* = −12→14
*T*_min_ = 0.761, *T*_max_ = 0.801	*k* = −20→21
24690 measured reflections	*l* = −25→25
4040 independent reflections	

### Refinement

**Table:**

Refinement on *F*^2^	Primary atom site location: difference Fourier map
Least-squares matrix: full	Secondary atom site location: difference Fourier map
*R*[*F*^2^ > 2σ(*F*^2^)] = 0.037	Hydrogen site location: inferred from neighbouring sites
*wR*(*F*^2^) = 0.102	H-atom parameters constrained
*S* = 1.03	*w* = 1/[σ^2^(*F*_o_^2^) + (0.0586*P*)^2^ + 1.2514*P*] where *P* = (*F*_o_^2^ + 2*F*_c_^2^)/3
4040 reflections	(Δ/σ)_max_ = 0.001
265 parameters	Δρ_max_ = 0.32 e Å^−^^3^
0 restraints	Δρ_min_ = −0.21 e Å^−^^3^

### Special details

**Table:**

Experimental. CrysAlisPro 1.171.41.117a (Rigaku OD, 2021) Empirical absorption correction using spherical harmonics, implemented in SCALE3 ABSPACK scaling algorithm.
Geometry. All esds (except the esd in the dihedral angle between two l.s. planes) are estimated using the full covariance matrix. The cell esds are taken into account individually in the estimation of esds in distances, angles and torsion angles; correlations between esds in cell parameters are only used when they are defined by crystal symmetry. An approximate (isotropic) treatment of cell esds is used for estimating esds involving l.s. planes.

### Fractional atomic coordinates and isotropic or equivalent isotropic displacement parameters (Å^2^)

**Table:**

	*x*	*y*	*z*	*U*_iso_*/*U*_eq_	
F1	0.46254 (7)	0.40849 (4)	0.69241 (4)	0.03490 (19)	
F2	0.47797 (8)	0.35558 (4)	0.59393 (4)	0.0394 (2)	
F3	0.62779 (7)	0.41697 (5)	0.63772 (5)	0.0411 (2)	
C1	0.25270 (10)	0.46229 (6)	0.61741 (6)	0.0228 (2)	
H1	0.2891	0.4136	0.6366	0.027*	
C2	0.18508 (10)	0.50270 (6)	0.67299 (6)	0.0234 (2)	
C3	0.06815 (11)	0.52036 (7)	0.68327 (6)	0.0269 (2)	
H3	0.0021	0.5056	0.6559	0.032*	
C4	0.06377 (11)	0.56583 (7)	0.74372 (6)	0.0287 (3)	
H4	−0.0059	0.5875	0.7641	0.034*	
C5	0.17741 (11)	0.57194 (7)	0.76607 (6)	0.0264 (2)	
C6	0.23342 (11)	0.61600 (7)	0.82269 (6)	0.0291 (3)	
H6A	0.1701	0.6439	0.8481	0.035*	
H6B	0.2730	0.5786	0.8536	0.035*	
N7	0.32220 (10)	0.67275 (6)	0.79832 (5)	0.0295 (2)	
C8	0.27337 (13)	0.73574 (7)	0.75550 (7)	0.0337 (3)	
H8A	0.2940	0.7876	0.7747	0.040*	
H8B	0.1848	0.7315	0.7532	0.040*	
C9	0.32582 (12)	0.72806 (7)	0.68700 (6)	0.0299 (3)	
C10	0.42408 (12)	0.75661 (7)	0.65530 (7)	0.0318 (3)	
H10	0.4722	0.7997	0.6692	0.038*	
C11	0.44143 (11)	0.70908 (7)	0.59651 (6)	0.0286 (3)	
H11	0.5035	0.7143	0.5641	0.034*	
C12	0.35196 (10)	0.65556 (7)	0.59651 (6)	0.0246 (2)	
C13	0.32223 (10)	0.58544 (6)	0.55457 (6)	0.0230 (2)	
H13	0.3733	0.5873	0.5133	0.028*	
C14	0.18934 (10)	0.58564 (6)	0.53217 (6)	0.0247 (2)	
H14	0.1393	0.5980	0.5725	0.030*	
C15	0.15409 (10)	0.50470 (7)	0.50819 (6)	0.0243 (2)	
O15	0.11156 (8)	0.49283 (5)	0.45311 (4)	0.0309 (2)	
C16	0.17334 (10)	0.43780 (7)	0.55769 (6)	0.0250 (2)	
H16	0.0930	0.4225	0.5759	0.030*	
N17	0.35219 (9)	0.51181 (5)	0.59182 (5)	0.0220 (2)	
C17	0.47033 (10)	0.49467 (7)	0.59476 (6)	0.0244 (2)	
O17	0.54942 (7)	0.53360 (5)	0.56885 (4)	0.0294 (2)	
O18	0.25418 (7)	0.53151 (5)	0.72431 (4)	0.02384 (18)	
C18	0.50906 (11)	0.41827 (7)	0.63123 (7)	0.0303 (3)	
O19	0.27850 (8)	0.66728 (5)	0.65050 (4)	0.02727 (19)	
C19	0.39990 (13)	0.70224 (8)	0.85115 (7)	0.0353 (3)	
H19A	0.4331	0.6581	0.8766	0.053*	
H19B	0.3534	0.7360	0.8813	0.053*	
H19C	0.4656	0.7325	0.8312	0.053*	
C20	0.16602 (12)	0.64922 (7)	0.48000 (7)	0.0320 (3)	
H20A	0.0798	0.6519	0.4704	0.048*	
H20B	0.2098	0.6368	0.4387	0.048*	
H20C	0.1934	0.6999	0.4974	0.048*	
C21	0.22552 (12)	0.36654 (7)	0.52092 (6)	0.0311 (3)	
H21A	0.1701	0.3501	0.4854	0.047*	
H21B	0.2367	0.3234	0.5528	0.047*	
H21C	0.3031	0.3806	0.5011	0.047*	

### Atomic displacement parameters (Å^2^)

**Table:**

	*U*^11^	*U*^22^	*U*^33^	*U*^12^	*U*^13^	*U*^23^
F1	0.0367 (4)	0.0339 (4)	0.0340 (4)	0.0041 (3)	−0.0030 (3)	0.0081 (3)
F2	0.0474 (5)	0.0240 (4)	0.0468 (5)	0.0084 (3)	−0.0041 (4)	−0.0032 (3)
F3	0.0281 (4)	0.0407 (4)	0.0544 (5)	0.0110 (3)	−0.0028 (3)	0.0045 (4)
C1	0.0233 (5)	0.0194 (5)	0.0258 (5)	−0.0018 (4)	0.0010 (4)	0.0013 (4)
C2	0.0253 (5)	0.0218 (5)	0.0231 (5)	−0.0035 (4)	−0.0007 (4)	0.0007 (4)
C3	0.0231 (5)	0.0307 (6)	0.0270 (6)	−0.0025 (4)	−0.0004 (4)	0.0010 (5)
C4	0.0246 (6)	0.0337 (6)	0.0279 (6)	0.0002 (5)	0.0034 (4)	−0.0007 (5)
C5	0.0270 (6)	0.0270 (6)	0.0253 (6)	−0.0001 (4)	0.0031 (4)	−0.0018 (4)
C6	0.0297 (6)	0.0311 (6)	0.0266 (6)	−0.0015 (5)	0.0011 (5)	−0.0028 (5)
N7	0.0329 (5)	0.0269 (5)	0.0288 (5)	−0.0024 (4)	−0.0017 (4)	−0.0021 (4)
C8	0.0418 (7)	0.0249 (6)	0.0345 (7)	0.0014 (5)	−0.0018 (5)	−0.0041 (5)
C9	0.0377 (7)	0.0207 (5)	0.0313 (6)	−0.0007 (5)	−0.0051 (5)	−0.0011 (4)
C10	0.0389 (7)	0.0211 (5)	0.0353 (7)	−0.0056 (5)	−0.0071 (5)	0.0025 (5)
C11	0.0300 (6)	0.0228 (5)	0.0330 (6)	−0.0038 (4)	−0.0018 (5)	0.0053 (5)
C12	0.0266 (5)	0.0205 (5)	0.0268 (5)	0.0005 (4)	−0.0016 (4)	0.0033 (4)
C13	0.0233 (5)	0.0200 (5)	0.0256 (5)	−0.0008 (4)	0.0005 (4)	0.0030 (4)
C14	0.0245 (5)	0.0228 (5)	0.0268 (6)	0.0003 (4)	−0.0011 (4)	0.0016 (4)
C15	0.0198 (5)	0.0262 (5)	0.0269 (6)	−0.0006 (4)	0.0016 (4)	−0.0010 (4)
O15	0.0305 (4)	0.0344 (5)	0.0278 (4)	−0.0005 (4)	−0.0040 (4)	−0.0028 (3)
C16	0.0263 (5)	0.0222 (5)	0.0265 (6)	−0.0048 (4)	0.0011 (4)	−0.0007 (4)
N17	0.0222 (5)	0.0185 (4)	0.0255 (5)	0.0001 (3)	0.0014 (4)	0.0017 (3)
C17	0.0236 (5)	0.0239 (5)	0.0258 (6)	0.0011 (4)	0.0004 (4)	−0.0019 (4)
O17	0.0234 (4)	0.0325 (4)	0.0322 (5)	−0.0013 (3)	0.0020 (3)	0.0012 (3)
O18	0.0223 (4)	0.0246 (4)	0.0246 (4)	−0.0010 (3)	0.0001 (3)	−0.0021 (3)
C18	0.0290 (6)	0.0270 (6)	0.0349 (6)	0.0049 (5)	−0.0005 (5)	0.0008 (5)
O19	0.0299 (4)	0.0235 (4)	0.0284 (4)	−0.0026 (3)	0.0005 (3)	−0.0017 (3)
C19	0.0393 (7)	0.0340 (6)	0.0326 (7)	−0.0059 (5)	−0.0025 (5)	−0.0057 (5)
C20	0.0332 (6)	0.0273 (6)	0.0355 (6)	0.0005 (5)	−0.0059 (5)	0.0059 (5)
C21	0.0407 (7)	0.0219 (5)	0.0307 (6)	−0.0039 (5)	0.0015 (5)	−0.0030 (5)

### Geometric parameters (Å, º)

**Table:**

F1—C18	1.3344 (15)	C11—C12	1.3511 (16)
F2—C18	1.3470 (15)	C11—H11	0.9500
F3—C18	1.3286 (15)	C12—O19	1.3655 (14)
C1—N17	1.4833 (14)	C12—C13	1.4958 (15)
C1—C2	1.5056 (16)	C13—N17	1.4961 (13)
C1—C16	1.5393 (16)	C13—C14	1.5455 (16)
C1—H1	1.0000	C13—H13	1.0000
C2—C3	1.3521 (17)	C14—C15	1.5125 (16)
C2—O18	1.3702 (14)	C14—C20	1.5239 (16)
C3—C4	1.4328 (17)	C14—H14	1.0000
C3—H3	0.9500	C15—O15	1.2117 (15)
C4—C5	1.3454 (17)	C15—C16	1.5230 (16)
C4—H4	0.9500	C16—C21	1.5333 (16)
C5—O18	1.3776 (14)	C16—H16	1.0000
C5—C6	1.4916 (16)	N17—C17	1.3489 (15)
C6—N7	1.4661 (16)	C17—O17	1.2176 (15)
C6—H6A	0.9900	C17—C18	1.5527 (16)
C6—H6B	0.9900	C19—H19A	0.9800
N7—C19	1.4520 (16)	C19—H19B	0.9800
N7—C8	1.4755 (17)	C19—H19C	0.9800
C8—C9	1.4895 (19)	C20—H20A	0.9800
C8—H8A	0.9900	C20—H20B	0.9800
C8—H8B	0.9900	C20—H20C	0.9800
C9—C10	1.3537 (19)	C21—H21A	0.9800
C9—O19	1.3713 (14)	C21—H21B	0.9800
C10—C11	1.4370 (18)	C21—H21C	0.9800
C10—H10	0.9500		
			
N17—C1—C2	111.43 (9)	N17—C13—H13	107.9
N17—C1—C16	108.54 (9)	C14—C13—H13	107.9
C2—C1—C16	113.89 (9)	C15—C14—C20	112.95 (10)
N17—C1—H1	107.6	C15—C14—C13	109.73 (9)
C2—C1—H1	107.6	C20—C14—C13	111.19 (10)
C16—C1—H1	107.6	C15—C14—H14	107.6
C3—C2—O18	110.37 (10)	C20—C14—H14	107.6
C3—C2—C1	134.01 (11)	C13—C14—H14	107.6
O18—C2—C1	115.57 (10)	O15—C15—C14	122.66 (11)
C2—C3—C4	106.29 (10)	O15—C15—C16	121.05 (10)
C2—C3—H3	126.9	C14—C15—C16	116.28 (10)
C4—C3—H3	126.9	C15—C16—C21	109.76 (10)
C5—C4—C3	106.73 (11)	C15—C16—C1	112.18 (9)
C5—C4—H4	126.6	C21—C16—C1	111.50 (10)
C3—C4—H4	126.6	C15—C16—H16	107.7
C4—C5—O18	110.19 (10)	C21—C16—H16	107.7
C4—C5—C6	133.02 (11)	C1—C16—H16	107.7
O18—C5—C6	116.68 (10)	C17—N17—C1	126.16 (9)
N7—C6—C5	111.36 (10)	C17—N17—C13	114.89 (9)
N7—C6—H6A	109.4	C1—N17—C13	118.79 (9)
C5—C6—H6A	109.4	O17—C17—N17	124.67 (11)
N7—C6—H6B	109.4	O17—C17—C18	117.06 (11)
C5—C6—H6B	109.4	N17—C17—C18	118.23 (10)
H6A—C6—H6B	108.0	C2—O18—C5	106.33 (9)
C19—N7—C6	113.00 (10)	F3—C18—F1	107.18 (11)
C19—N7—C8	112.71 (10)	F3—C18—F2	107.23 (10)
C6—N7—C8	115.05 (10)	F1—C18—F2	107.72 (10)
N7—C8—C9	108.70 (10)	F3—C18—C17	109.62 (10)
N7—C8—H8A	109.9	F1—C18—C17	115.10 (10)
C9—C8—H8A	109.9	F2—C18—C17	109.68 (10)
N7—C8—H8B	109.9	C12—O19—C9	107.32 (9)
C9—C8—H8B	109.9	N7—C19—H19A	109.5
H8A—C8—H8B	108.3	N7—C19—H19B	109.5
C10—C9—O19	109.59 (11)	H19A—C19—H19B	109.5
C10—C9—C8	135.42 (12)	N7—C19—H19C	109.5
O19—C9—C8	113.66 (11)	H19A—C19—H19C	109.5
C9—C10—C11	106.59 (11)	H19B—C19—H19C	109.5
C9—C10—H10	126.7	C14—C20—H20A	109.5
C11—C10—H10	126.7	C14—C20—H20B	109.5
C12—C11—C10	106.38 (11)	H20A—C20—H20B	109.5
C12—C11—H11	126.8	C14—C20—H20C	109.5
C10—C11—H11	126.8	H20A—C20—H20C	109.5
C11—C12—O19	110.05 (10)	H20B—C20—H20C	109.5
C11—C12—C13	134.69 (11)	C16—C21—H21A	109.5
O19—C12—C13	115.09 (10)	C16—C21—H21B	109.5
C12—C13—N17	110.17 (9)	H21A—C21—H21B	109.5
C12—C13—C14	111.80 (9)	C16—C21—H21C	109.5
N17—C13—C14	111.01 (9)	H21A—C21—H21C	109.5
C12—C13—H13	107.9	H21B—C21—H21C	109.5
			
N17—C1—C2—C3	125.68 (14)	O15—C15—C16—C21	42.86 (15)
C16—C1—C2—C3	2.49 (18)	C14—C15—C16—C21	−137.91 (10)
N17—C1—C2—O18	−51.30 (13)	O15—C15—C16—C1	167.40 (11)
C16—C1—C2—O18	−174.48 (9)	C14—C15—C16—C1	−13.37 (14)
O18—C2—C3—C4	2.27 (13)	N17—C1—C16—C15	−40.88 (12)
C1—C2—C3—C4	−174.82 (12)	C2—C1—C16—C15	83.87 (12)
C2—C3—C4—C5	−0.54 (14)	N17—C1—C16—C21	82.69 (11)
C3—C4—C5—O18	−1.37 (14)	C2—C1—C16—C21	−152.56 (10)
C3—C4—C5—C6	174.71 (13)	C2—C1—N17—C17	116.85 (12)
C4—C5—C6—N7	−118.73 (15)	C16—C1—N17—C17	−116.96 (12)
O18—C5—C6—N7	57.15 (14)	C2—C1—N17—C13	−67.88 (12)
C5—C6—N7—C19	−165.25 (11)	C16—C1—N17—C13	58.31 (12)
C5—C6—N7—C8	63.35 (13)	C12—C13—N17—C17	−75.59 (12)
C19—N7—C8—C9	112.65 (12)	C14—C13—N17—C17	160.03 (10)
C6—N7—C8—C9	−115.81 (12)	C12—C13—N17—C1	108.62 (11)
N7—C8—C9—C10	−90.45 (17)	C14—C13—N17—C1	−15.77 (13)
N7—C8—C9—O19	74.55 (13)	C1—N17—C17—O17	174.16 (11)
O19—C9—C10—C11	−1.95 (14)	C13—N17—C17—O17	−1.27 (17)
C8—C9—C10—C11	163.48 (14)	C1—N17—C17—C18	−3.30 (17)
C9—C10—C11—C12	0.47 (14)	C13—N17—C17—C18	−178.73 (10)
C10—C11—C12—O19	1.19 (13)	C3—C2—O18—C5	−3.10 (12)
C10—C11—C12—C13	−173.65 (12)	C1—C2—O18—C5	174.59 (9)
C11—C12—C13—N17	103.88 (15)	C4—C5—O18—C2	2.73 (13)
O19—C12—C13—N17	−70.77 (12)	C6—C5—O18—C2	−174.06 (10)
C11—C12—C13—C14	−132.19 (14)	O17—C17—C18—F3	11.43 (15)
O19—C12—C13—C14	53.16 (13)	N17—C17—C18—F3	−170.92 (10)
C12—C13—C14—C15	−163.52 (9)	O17—C17—C18—F1	132.31 (12)
N17—C13—C14—C15	−40.06 (12)	N17—C17—C18—F1	−50.03 (15)
C12—C13—C14—C20	70.80 (12)	O17—C17—C18—F2	−106.05 (12)
N17—C13—C14—C20	−165.74 (10)	N17—C17—C18—F2	71.60 (14)
C20—C14—C15—O15	−0.18 (16)	C11—C12—O19—C9	−2.39 (13)
C13—C14—C15—O15	−124.85 (12)	C13—C12—O19—C9	173.57 (9)
C20—C14—C15—C16	−179.39 (10)	C10—C9—O19—C12	2.69 (13)
C13—C14—C15—C16	55.94 (13)	C8—C9—O19—C12	−166.19 (10)

### Hydrogen-bond geometry (Å, º)

*Cg*2 is the centroid of the O19/C9–C12 ring.

**Table:**

*D*—H···*A*	*D*—H	H···*A*	*D*···*A*	*D*—H···*A*
C1—H1···F1	1.00	2.23	2.9210 (14)	125
C1—H1···F2	1.00	2.47	3.1341 (14)	123
C3—H3···O15^i^	0.95	2.51	3.3809 (15)	152
C14—H14···O19	1.00	2.49	2.9114 (14)	105
C20—H20*B*···F3^ii^	0.98	2.54	3.4700 (16)	160
C21—H21*B*···*Cg*2^iii^	0.98	2.88	3.7561 (13)	150

Symmetry codes: (i) −*x*, −*y*+1, −*z*+1; (ii) −*x*+1, −*y*+1, −*z*+1; (iii) −*x*+1/2, *y*−1/2, *z*.
